# Bacteriophages against *Serratia* as Fish Spoilage Control Technology

**DOI:** 10.3389/fmicb.2017.00449

**Published:** 2017-04-04

**Authors:** Igor Hernández

**Affiliations:** AZTI-Tecnalia, Food Research Division, Parque Tecnológico de Bizkaia,Derio, Spain

**Keywords:** phagotherapy, *Serratia*, fish spoilage, shelf life, *Trachurus trachurus*

## Abstract

Bacteria of the genus *Serratia*, mainly *S. proteamaculans* and *S. fonticola*, are important spoilage agents in Atlantic horse mackerel (*Trachurus trachurus*). In order to evaluate whether bacteriophages against *Serratia* could delay the spoilage process, 11 viral strains active against this genus were isolated from food and best candidate was applied to fresh mackerel filets. All the phages belong to the *Siphoviridae* and *Podoviridae* families and were active at multiplicity of infection (MOI) levels below 1:1 in Long & Hammer broth. The ability of phage AZT6 to control *Serratia* populations in real food was tested in Atlantic horse mackerel extract and applied to fresh mackerel filets. Treatment with high phage concentration (MOI 350:1, initial *Serratia* population 3.9 ± 0.3 Log cfu/g) can reduce the *Serratia* populations up to 90% during fish storage (a maximum of 6 days) at low temperatures (6°C). Bacterial inhibition was dependent on the bacteriophage dosage, and MOI of 10:1 or lower did not significantly affect the *Serratia* populations.

## Introduction

Bacterial viruses (bacteriophages) are considered to be the most abundant organisms on the planet. Their ability to reduce bacterial loads has led them to be suggested as therapies for human and animal microbial infections (Brüssow, 2012), but other applications are also possible. In food technology, phages have been proposed for food spoilage control (García et al., 2008; Mahony et al., 2011), and selected phages have been tested in food contaminated with *Salmonella* (Leverentz et al., 2004), *Listeria* (Leverentz et al., 2004; Carlton et al., 2005), or *Escherichia coli* (Carter et al., 2012). However, few references describe phages as a strategy against spoilage bacteria for shelf life extension of food. Bacteriophages against spoilage bacteria have the advantage that natural resistance of bacteria to lysis by phage is not a critical safety concern, while even modest reduction of specific microorganisms can significantly prolong food’s shelf life. To the best of our knowledge, the first reference of phages as food “spoilage control agents” was from Ellis et al. (1973), who worked on milk storage. Later, other authors described phages that were active against *Pseudomonas* in beef (Greer and Dilts, 1990) and in milk (Sillankorva et al., 2008), *Listeria brevis* in beer (Deasy et al., 2011*)*, *Brochothrix thermosphacta* in pork (Greer and Dilts, 2002), and *Shewanella* in fish (Li et al., 2014). In most cases, the target bacteria were inoculated in the food matrix, and the activity against naturally presenting bacteria was not considered; hence, complicates the transfer of the technology to the food industry.

In previous work, Alfaro and Hernández (2013) determined that *Serratia* is a dominant bacterial genus (over 25% of the total bacterial load) at sensorial rejection time of Atlantic Horse Mackerel (*Trachurus trachurus*) filets stored in modified atmospheres (MAPs), and the increase of these bacteria during fish storage has been related to their fresh shelf life (Alfaro et al., 2013). Many bacterial species have been associated with formation of biogenic amines (De Filippis et al., 2013), and they are part of the spoilage microflora of diverse foods (Doulgeraki et al., 2012). The *Serratia* genus are Gram negative, facultative anaerobic bacteria, which grow in a broad range of temperatures and substrates, including plant surfaces, soil, water, and food products such as fruit juices and fish (Garrity et al., 2005). Many species have been related with food spoilage, and some of them (mainly *S. marcescens* and *S. liquefaciens*) have been described as opportunistic human pathogens (Mahlen, 2011).

Bacteriophages for *Serratia* have been purified before from sewage effluent, mainly using strains of *S. marcescens* as targets. Those virons have a relatively wide broad range of action, and are even able to infect bacteria of genera other than *Serratia* (Prinsloo and Coetzee, 1964; Prinsloo, 1966; Evans et al., 2010). Phages against *Serratia* have been used as molecular biology tools (Petty et al., 2006) and as models for viron abundance estimation in soil (Ashelford et al., 1999, 2003). To the best of our knowledge, no utilization in the food industry has been reported.

This paper describes the isolation, selection, and characterization of bacteriophages against *Serratia* species, and the evaluation of their ability to control growth of naturally occurring *Serratia* during spoilage of Mackerel filets. The research reported here provides an example of the potential and limitations of the application of bacteriophages to retard bacterial spoilage in foods.

## Materials and Methods

### Bacterial Strains and Culture Media

*Serratia fonticola* and *S. proteamaculans* strains were isolated in our facilities from spoiling Atlantic horse mackerel and were characterized by 16S RNA sequencing as previously described (Alfaro and Hernández, 2013). Two strains of *S. fonticola* (SFO001 and SFO002) and six strains of *S. proteamaculans* (SPR001, SPR002, SPR004, SPR005, SPR006, and SPR009) were used for phage isolation and characterization. Two *S. marcescens* strains (CECT 854 and CECT 977) were purchased from the Spanish type culture collection (CECT).

Long & Hammer agar and Long & Hammer broth (Koutsoumanis and Nychas, 1999) were used as general propagation media for *Serratia*. Strains were cultivated for 24 h at room temperature (22–25°C) in aerobic conditions. Bacterial populations in fish samples were quantified using: (i) *Serratia* selective agar (SSA) prepared as described by Starr et al. (1976) for enumeration of *Serratia.* Incubations were aerobic at 20°C. (ii) Triptone soya agar (TSA) incubated aerobically at 30°C for 24 h for total bacterial counts. (iii) Long & Hammer agar incubated aerobically at 12°C for 72 h for psychrotropic total bacterial counts. Peptone water was used for decimal dilutions.

Spoilage-related bacteria previously isolated and characterized from Atlantic horse mackerel (Alfaro and Hernández, 2013) were used to study the phage specificities. *Carnobacterium maltaromaticum*, *Shewanella putrefaciens. Vibrio* ssp., and *Yersinia intermedia* strains were used for phage specificity determinations. Bacteria in exponential growth phase (in TSB) were treated with phages of interest (MOI 1000:1), and growth was controlled by optical density at 600 nm (OD_600__nm_) and by colony count in TSA (incubated at 30°C for 24 h).

### Phage Isolation and Enumeration

The *Serratia* bacteriophages were isolated from cheese whey obtained from artisanal cheese producers. Briefly, whey samples were mixed with an exponential (OD_600_ = 0.250–0.300) growth culture of SFO001 (whey:culture: 1:1) and incubated at 20°C for 7 h. These phage-rich samples were mixed with chloroform (10:1 v/v), centrifuged (3 min, 14.000 × *g*) and the water fractions were transferred to sterile tubes. Water fractions (or their decimal dilutions) were mixed (1:1) with high-density SFO001 culture (OD = 1.5) and with Long & Hammer soft agar. The mixture was smeared over a plate of Long & Hammer agar and incubated at 20°C for 24 h until plaques were visible. For further purifications, one plaque from each plate was extracted, dissolved in peptone water and treated as described above for whey samples. Three purification cycles were performed for each phage.

Phage enumeration was done with a spot test and double-layer agar technique (Kropinski et al., 2009; Mazzocco et al., 2009), using exponentially growing SFO001 as sensible strains. Plates (Long & Hammer agar and soft agar) were incubated at 25°C for 24 h. Samples of 10 μl of phage dilution were used for the spot tests. Phage dilutions were done in sterile saline–magnesium–gelatine buffer (SGM, 0.1 M NaCl, 1 mM MgSO_4_^∗^7H_2_O, 50 mM Tris–HCl and 0.01% gelatine, pH = 7.5).

SPR009 has a high growth rate at a broad-range of growth temperatures, which facilities the use of this strain for phage-activity characterization in liquid medium. Long & Hammer broth was used.

For stock production of phages, they were cultivated with exponentially growing target strain (SPR009) in fresh Long & Hammer broth at 20°C for 24 h. Bacteria were hydrolyzed and removed by mixing the culture with chloroform (10:1), vortex and centrifuged (5 min, 25000* × g*). Supernatant was spread on a Petri plate under sterile air for 15 min and stored at 4°C or at -80°C (20% glycerol added) until used. When high volumes of bacteriophage were necessary, samples were purified and concentrated as described by Sambrook and Russell (2001), using modified Long & Hammer broth (gelatin concentration was reduced to 1% and 10 mM CaCl_2_ was added) as cultivation medium and PEG6000 as precipitation agent.

### Phage Characterization

For molecular characterization, DNA was isolated according to the method described by Sambrook and Russell (2001). For restriction endonuclease analyses, DNA was digested with EcoRI (New England Biolabs) according to the manufacturer’s recommendations. Fragment lengths were verified with gel electrophoresis (0.8% agarose in TAE buffer, 70v; Biorad, Madrid, Spain) and visualized in a BioDoc-it imaging system (UVP, Upland, CA, USA). Generuler 1-kb DNA leader was used as a calibration standard (VWR, Madrid).

Phage morphology was studied by transmission electron microscopy (TEM). For purification, phage dilution (estimated as 10^12^ pfu/ml) was centrifuged (1500* × g*, 60 min, 4°C), and the pellet was dissolved in sodium acetate solution. Centrifugation was repeated, and the pellet was finally dissolved in sodium acetate. For sample preparation, 5 μl of phage dilution was fixed in an Agar S-160 carbon rack temporally hydrophilized by glow discharge (20 s, 800v DV in vacuum conditions). Phages were stained with uranyl acetate 1% (twice, 30 s each). Phage morphology was examined with TEM (Philips CM120 biofilter) and photographed with an Olympus SIS “Morada” camera. Phage morphology and dimensions (capsid diameter, tail length, and width) were analyzed from electron micrographs with ImageJ software (Rasband, Ver 1.48).

Phage lytic spectra were determined using fresh phage propagated in SFO001 and double agar layer technique as described above. Plaques were counted after 24 h at 25°C.

### Evaluation of Phage Activity in Fish Broth and Filets

For fish broth preparation, five fresh Atlantic horse mackerel were eviscerated, cut in small pieces and mixed. Then 1 kg of the fish was boiled for 10 min in 1.0 l of distilled water. The broth was filtered through 1 mm pores and autoclaved 15 min at 110°C. Fresh inoculum of strain SPR009 was used to inoculate the broth (final concentration (3.9 ± 0.2 log cfu/ml) and AZT6 phage was inoculated 10 min later at 6 or 20°C. After 5 days, a 1 ml sample was removed for bacterial and phage concentration determinations. Bacterial load was estimated on Long & Hammer agar (25°C, 24–48 h), and phage load was determined by the spot-test technique described above. Each experimental condition was studied in triplicate.

For antimicrobial activity on fish filets, *T. trachurus* filets were used as a model food matrix. These filets were not inoculated with bacteria, and the results reflect reduction of natural microbiota. Two fresh fish were supplied by each of two different local markets, eviscerated, heads-removed under sterile conditions and the bodies divided along the vertebral bones in two similar portions (in total eight “filets”). Four filets were submerged for 60 s in AZT6 phage solution (SMG buffer with different concentrations of phages), dried for 60 s under sterile air and stored in aseptic 140-mm Petri plates with plastic covers. Samples were stored aerobically at 6°C in a temperature-controlled chamber. After 3 or 6 days at 6°C, 5–10 g of fish from each filet were aseptically removed, homogenized in APT (fish:broth ratio was 1:10) using a Stomacher^®^ blender (6 ×{20′′ on and 10′′ off}) and used for bacterial counts.

### Statistical Analysis

PSPP V0.6.2 (Free Software Foundation, Inc.) was used for analysis of variance (ANOVA) and least significant difference (LSD) statistical procedures. A confidence interval of 95% (*p* ≤ 0.05) was used.

## Results and Discussion

### Phage Isolation and Characterization

Working with *Serratia fonticola* SFO001 the as target strain, 11 phages were isolated from cheese whey samples using the double-agar layer technique. Initial phage purification did not require Ca^2+^ addition, but addition of 0.5 mM Ca^2+^ increased the plaque size after the last purification cycle.

Isolated phages consistently produced clear plaques on Long & Hammer agar inoculated with *Serratia* SFO001 and, therefore, the whey phages were classified as virulent. Analysis showed that DNA of all of the isolated virons was double stranded and produced different DNA digestion profiles with EcoRI (results not shown). We discarded duplicate isolations. Estimated DNA size ranged from 50 to 70 Kbp.

**FIGURE 1:**
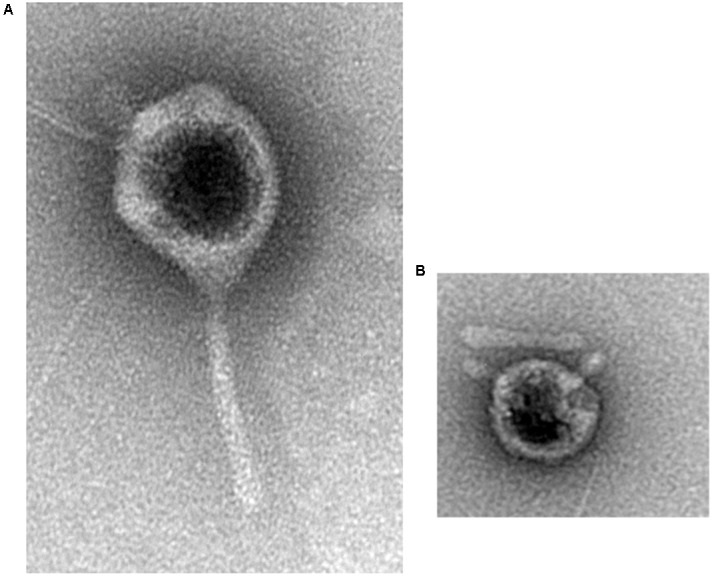
**Transmission electron micrographs of AZT6 (A)** and AZT4 **(B)**.

Phages AZT1, AZT2, AZT3, AZT5, AZT6, and AZT9 have icosahedral heads (80–90 nm) and long non-contractile tails (100–120 nm). In contrast, AZT4, AZT7, AZT8, AZT10, and AZT11 presented spherical heads (75–90 nm) and extremely short tails (10–15 nm). All the isolated virons have lytic activity after 10 min of incubation in chloroform, suggesting that a lipid envelope is not a functional requirement. The first group would be classified as type B in Bradley’s classification (Bradley, 1967), and they probably belong to the *Siphoviridae* family. The second group would be C-type phages (Bradley, 1967) classified with the *Podoviridae*. **Figure 1** presents the typical conformation of each type of phage. Other authors have demonstrated the highly variable morphology of the *Serratia* phages, even when they are isolated from the same source (Bradley, 1965; Ashelford et al., 2003).

Maximal phage titrations in the medium after a single cultivation round were between 6.9 and 9.3 Log pfu/ml (Supplementary Table 1). All bacteriophages were active after 30 days at -80°C with losses of viability less than 2.0 Log pfu/ml, except AZT5 and AZT8 that lost viability by more than five orders of magnitude during ultrafreezing. Similar activity losses in pure phage solutions were observed after 30 days at 4°C (Supplementary Table 2).

**FIGURE 2:**
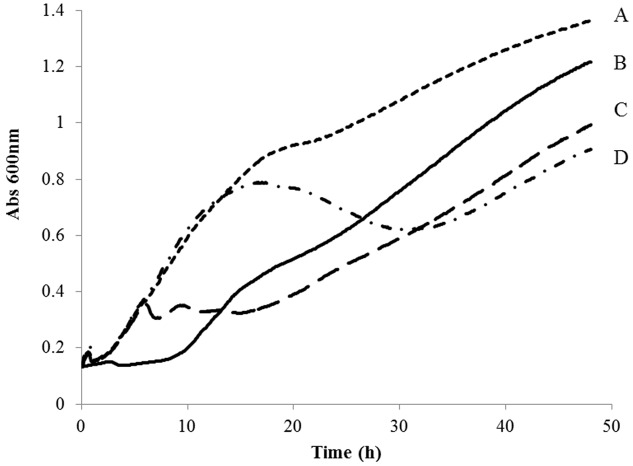
**Optical density (OD_600_) evolution of strain SPR009 cultivated in Long & Hammer broth at 20°C treated with cultures AZT3 at different MOI. (A)** No phages, **(B)** 100:1, **(C)** 1:1, **(D)** 1:1000.

Bactericidal effects depend on MOI and, in most of the cases, effective MOI at 20°C ranged over five orders of magnitude (**Figure 2**, AZT3 as an example). An MOI close to 100:1 produced an initial decrease in bacterial concentration, followed by a normal growth curve. Lower phage doses (when activity is detected) permit initial bacteria growth, followed by decreases in bacterial concentration in later phases (**Figure 2**). In all cases, MOI of 1:1 or higher resulted in significant bacterial reduction (i.e., Supplementary Figure 1 for MOI 1:1) and doses with MOI smaller than 1:1000 were not tested. In most cases, no significant differences were observed in the phage activity while varying calcium concentration (up to 10 mM Ca^2+^, Supplementary Figure 1) in broth. However, significant increases of inhibitory activity were observed using phages AZT7 and AZT9 when Ca^2+^ was increased over this concentration (results not shown).

Lytic spectrum (**Table 1**) showed high diversity among isolated phages. AZT6 showed the highest activity against four strains, while AZT5 is specific for SFO001 and AZT1 and AZT8 have a narrow activity range. None of the phages were active against all the considered strains.

**Table 1:** Lytic spectra of isolated phages against considered bacterial strains.

		Considered phage^a^								
Bacterial species	Strain	AZT1	AZT2	AZT3	AZT4	AZT5	AZT6	AZT7	AZT8	AZT9
*S. fonticola*	SFO001	+++^b^	+++	+++	+++	+++	+++	+++	+++	+++
	SFO002	−	+	−	+	−	++	−	−	−
*S. proteamaculans*	SPR001	+	−	++	−	−	+++	+	−	++
	SPR002	−	−	−	−	−	−	−	−	−
	SPR004	−	+++	−	−	−	−	−	−	−
	SPR005	−	−	+	−	−	−	−	−	++
	SPR006	−	++	−	−	−	+++	−	++	−
	SPR009	−	−	−	−	−	+++	−	−	−
*S. marcensens*	CECT854	−	−	−	++	−	−	−	−	−
	CECT977	−	−	−	−	−	−	−	−	++

*^a^Phage total count was 2 × 10^6^ pfu/ml using SFO001 as sensible strain.*

*^b^+++, > 10^5^ pfu/ml; ++ 10^3^ to 10^5^ pfu/ml; +, 10^1^ to 10^3^ pfu/ml; -, no visible calves.*

Two phages were active against *S. marcescens* strains. AZT9 reduced populations of CECT977 by 35%, and AZT4 reduced those of CECT854 by 46% when the bacteria were grown in TSB supplemented with 2 mM Mg^2+^ and 2 mM Ca^2+^. That lytic activity was low, so those phages have only weak impacts on the tested *S. marcescens* strains. None of the isolated phages were active against both strains.

Only AZT6 was selected for further studies. Reasons to discard other isolations include quick recovery of maximal growth rate after treatment (phages AZT1, AZT2, and AZT3), no activity at low MOI (AZT4 and AZT9), low stability at -80°C (AZT5 and AZT8) and maximal titrations below 8.5 Log pfu/ml (AZT7, AZT10, and AZT11).

### AZT6 Characteristics

Four strains of *C. maltaromaticum, S. putrefaciens, Y. intermedia* and *Vibrio* spp., isolated in our lab from spoiled fishes (Alfaro and Hernández, 2013), were treated at high MOI to test AZT6 phage specificity. For all strains of these bacteria, our results (not shown) showed that phage-treated and -untreated samples had similar kinetic growth parameters (Lag phase duration, growth rate, maximal yield). As expected, AZT6 was specific for *Serratia* and did not affect other genera of spoilage bacteria. AZT6 has a broad host range and produced lytic calves with both *S. fonticola* (SFO001 and SFO002) and *three S. proteamaculans* (SPR001, SPR006, and SPR009; **Table 1**).

AZT6 reduced the growth rate and delayed the stationary phase of SPR009 when that strain was cultivated between 4 and 20°C (MOI 1:1, results not shown), demonstrating activity in the temperature range of food storage. At higher temperatures, bactericidal effects disappeared after the initial 24 h, when bacteria had reached to stationary phase, but 48 h or longer was necessary to verify bacterial growth reduction at low temperatures.

Cultivation broth had an important effect on phage activity. In clear medium (Long & Hammer) MOI of 1:500 or higher produced significant reduction in bacterial counts, but in fish broth, an MOI of 1:3 was required to reduce growth of the *Serratia* population. Along with other factors, fish extract presents high numbers of particles in suspension that would reduce the activity of the bacteriophages.

Phage titration of AZT6 was stable during storage at cold temperatures. In pure culture, phage concentration was reduced 10-fold in the first 30 days at 6°C (from 8.9 ± 0.0 to 7.9 ± 0.2 Log pfu/ml), after which constant titration lasted up to 6 months at 4°C (from 7.9 ± 0.2 to 7.7 ± 0.2 Log pfu/ml). Ultra-cold storage (-80°C, 30 days) in the presence of glycine (20%) resulted in a titration reduction of an order of magnitude.

### Phage Activity in Liquid Medium

In order to select the best strategy for increasing the shelf life of fish products stored at 6°C, different MOI were tested in Long & Hammer broth. As observed in **Table 2**, MOI of 20:1 or higher have a high impact on bacterial load during initial storage (1–3 days), but then their effect decreased up to day 7, when no reduction was observed. Lower MOI did not initially reduce the bacterial growth or have a significant effect after three or more storage days. These results would agree with the expected mode of action of bacteriophages: at high concentration, bacteriophages cause initially a high bacterial decrees and days after treatment, phage-resistant bacteria growth rapidly. In the other hand, low phage dosage requires some time to propagate the infection and to reduce bacterial count significantly.

**Table 2:** Bacterial counts reduction (control – AZT6 treated samples, strain SPR009; Log cfu/ml) in Long & Hammer medium stored at 6°C using different MOI.

Time (days)	MOI			
	20:1	1:1	1:5	1:500
0	0.8^∗^	0.0	0.0	0.0
1	0.4^∗^	0.1	0.0	0.0
2	0.8^∗^	0.8^∗^	0.3	-0.1
3	1.1^∗^	1.4^∗^	1.3^∗^	0.6^∗^
4	0.3	0.8^∗^	0.8^∗^	0.6^∗^
7	0.1	0.4	0.6^∗^	1.0^∗^

*^∗^Significant differences between treated and control samples (p < 0.05) at different sampling times.*

Phage activity under real fish-storage conditions (6°C) was tested in fish extract medium using SPR009 as the sensible strain. High phage:bacteria ratios significant reduced the bacterial concentration, up to 99.9% of inhibition after 5 days (**Table 3**). Use of a MOI lower than 1:1 had no effect on bacterial concentration at the tested storage times. Addition of Ca^2+^ (up to 5 mM) to the fish extract did not affect phage activity (results not shown), similar to the results obtained in Long & Hammer broth.

**Table 3:** Bacterial counts reduction (control – AZT6 treated samples, strain SPR009, Log cfu/ml) in fish extract stored at 6°C for 5 days using different initial AZT6 and bacterial concentrations.

Initial bacteria (Log/ml)	Initial phage (Log/ml)	MOI (phag:bact)	Bacteria reduction^∗^ (Log cfu/ml)	Bacterial reduction^∗^ (%)
3.9	4.4	3:1	1.6	97.5
4.6	5.5	8:1	2.1	99.4
3.9	5.4	30:1	1.0	90.0
4.6	6.5	80:1	1.7	97.8
3.6	6.0	250:1	2.0	99.0
3.6	7.0	2500:1	2.9	99.9

*^∗^Bacterial reduction using the untreated samples as controls. In all cases, differences are statistically significant.*

### Phage Activity in Fish Filets

Determine the specific counts of *Serratia* in a wild environment represented an analytical problem in this research. Medium SSA has been described as selective for *Serratia* (Starr et al., 1976) and does not allow the growth of other representative strains of major bacterial genera in spoiled *T. trachurus*: *Yersinia, Photobacterium, Vibrio, Shewanella*, and *Carnobacterium*. Only *Serratia* formed visible colonies in that medium (results not shown), which supports that counts in SSA mainly were *Serratia*. Nevertheless, we cannot fully discount that other bacteria might grow in this medium.

Phage AZT6 was effective when applied to raw fish filets, and it can reduce of the *Serratia* load after 6 days by more than 90%. When fresh fish filets were treated with high phage–concentration solutions (7.8 Log pfu/g, MOI 350:1) results showed a significant reduction of bacterial counts in SSA after 3 (-0.4 Log cfu/g) and six storage days (-1.1 Log cfu/g) in treated samples compared to control samples (**Table 4**, bach 1).

**Table 4:** Bacterial concentrations (Log cfu/g) in fish filets treated (MOI 350:1) or not with AZT6 after 3 and 6 days at 6°C.

		Day 3		Day 6	
Bach	Medium	Treated	Not treated	Treated	Not treated
1	SSA	5.3 ± 0.1^∗^	5.7 ± 0.0	5.2 ± 0.1^∗^	6.3 ± 0.3
	TSA	7.0 ± 0.0^∗^	7.7 ± 0.2	7.3 ± 0.1^∗^	8.1 ± 0.1
	L & H	7.4 ± 0.1^∗^	8.0 ± 0.2	8.0 ± 0.1	8.1 ± 0.1
2	SSA	3.7 ± 0.2^∗^	5.2 ± 0.1	6.3 ± 0.3	6.5 ± 0.3
	TSA	7.7 ± 0.4	7.0 ± 0.4	8.6 ± 0.4	8.7 ± 0.4
	L & H	8.1 ± 0.4	7.5 ± 0.4	9.1 ± 0.4	9.2 ± 0.4

*^∗^Significant differences between treated and untreated fish filets (p < 0.05) at each sampling times.*

As expected, reduction in *Serratia* counts in treated filet samples compared with untreated ones were parallel to significant reductions (*p* < 0.05) of total bacterial counts after 3 and 6 days in TSA agar (-0.7 and -0.8 Log cfu/g respectively) and after 3 days (0.6 Log cfu/g) in Long & Hammer agar (**Table 4**, bach 1). In this medium, no differences were observed after 6 days, probably because bacterial counts had reached their maxima. Other bacteria genus, like *Yersinia, Photobacterium* and *Carnobacterium*, are counted in Long & Hammer agar and have been identified as important spoilage bacteria in *T. trachurus* (Alfaro and Hernández, 2013). These genera can grow vigorously in refrigeration (Alfaro et al., 2013) and, presumably, compensate the decrease in *Serratia* counts. A combination of bacteriophages active against different species would be a good strategy to reduce total bacteria and to increase the product’s shelf life. To the best of our knowledge, no examples of bacteriophage mixture against different genera in food have been published, but the concept has been proved successfully in pharmacology (Chan and Abedon, 2012) and further research would validate this strategy as food preservation technique.

When fish filets from other batches were treated with two phage dosages (MOI 100:1 or 30:1), the one with the highest phage:bacteria ratio showed significantly lower *Serratia* concentration after 3 days (-1.5 Log cfu/g), but loads were comparable after six spoilage days (**Table 4**, bach 2). These dosage effect differences were not detected in TSA or Long & Hammer agar (**Table 4**, bach 2).

**FIGURE 3:**
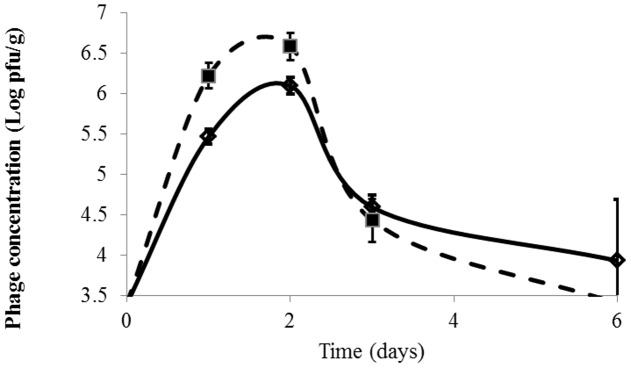
**Evolution of phage concentration (Log pfu/ml) in fish tissue during fish spoilage at 6°C.** Fish 1 and Fish 2. Detection limit = 3.5 Log pfu/g. Phage concentatrion in not inoculated samples were always below 3.5 Log pfu/g. Initial value (time = 0) is <3.5 Log pfu/g. Visible initial point is 0.4 h after phage application.

Nevertheless, no count reductions similar to those in SSA, TSA, or Long & Hammer agar were observed in the filets treated with phages at MOI ≤ 10:1 (results not shown), even in the initial days. Phage AZT6 was active in broth at low MOI, and other researchers have described experience with phages active at low MOI (1:1) in pork adipose tissue (Greer and Dilts, 2002), but AZT6 required an MOI higher than 10:1, as have other phages described in the literature (Guenther and Loessner, 2011). It can be speculated that different strains of *Serratia* active on different fish samples could modify the bacterial response to phage treatments, that AZT6 is not able to multiply in or remain stable in the mackerel food matrix. As can be observed in **Figure 3**, when MOI ≤ 10:1 were used, AZT6 concentration in fish muscle increased in the first 2 days, and then decreased along fish spoilage; phage titrations went from the initial 5.9 Log pfu/ml to near the detection limit after 6 days. AZT6 titration in buffer solution was stable under the experimental conditions (time, temperature, pH), and no a clear cause was found to explain the decreases on the filets. Stability of other phages tested in food matrices were dependent on the food pH (Leverentz et al., 2001) or, as we expected for AZT6, they were stable in food matrices throughout storage (Guenther et al., 2009). Phages can be stabilized by absorbing them into water–base matrix (Murthy and Engelhardt, 2012) or microencapsulated (Ma et al., 2008; Dini et al., 2012), that would result in a controlled bacteriophage release.

Phage adherence and penetration in fish samples are fast. Just 25 min after immersing fish filets in an AZT6 solution at 7.9 Log pfu/ml, we observed that phage concentration in the muscle increased from an initial value below 3.5–5.9 Log pfu/g (**Figure 3**). Phage AZT6 that settled on the filet surface penetrated by passive diffusion into the fish muscle that seems to be a fast and efficient process.

Our first attempt to test the phage activity in a solid matrix was to inoculate sterile fish cubes (autoclaved at 121°C for 15 min) with phage-sensitive SFO001 *Serratia* strain, and then treating them with high phage MOI (1000:1). After 24 and 96 h at 6°C, there was no bacteriophage effect on bacterial counts (results not shown). It is well-known that phage activity is favored in liquid media, and changes in muscle texture and reduction in food moisture would reduce phage activity. Phages are not motile organisms and their distributions are strongly dependent on conditions in food matrices. That is why most reported uses of bacteriophage involve adding them to liquid foods, like beer (Deasy et al., 2011) or milk (Modi et al., 2001; García et al., 2007). In only a few cases have they been shown to act on food surfaces (Guenther and Loessner, 2011).

Very few papers have been published about using phages for biocontrol of unintentionally contaminated foods. Probably, the report most similar to our study is that by Greer and Dilts (1990), who reported the inability of phages to control the spoilage of beef by wild *Pseudomonas*. The results presented in this paper not only demonstrate that the reduction of *Serratia* populations during spoilage is possible, but also demonstrated that many factors have to be adjusted for useful effective applications. Natural resistance is considered a problem for industrial applications. A mixture of phages providing broad spectra toward multiple target strains could to be an effective strategy. Another strategy would be that used by García et al. (2007), who transformed phages against *Staphylococcus aureus*, obtaining mutant phages that reduced otherwise bacteriophage-insensitive *S. aureus* variant levels up to 200-fold. A similar strategy would be useful for bacteriophage development against spoilage bacteria. Other factors, like application duration, have been reported as critical for effective phage activity in food (Leverentz et al., 2004).

We have described here a preliminary strategy for effective attenuation of *Serratia* growth during fish spoilage. Phage AZT6 is a promising candidate for application in order to reduce the *Serratia* population in freshly butchered fish tissues. It is active at low MOI, can be produced at high titration, and is stable for long periods under refrigeration. It shows bactericidal activity for multiple *Serratia* species, and we demonstrated its activity in real food matrices subject to spoilage. When MOI was 350:1, the bactericidal effect was up to 90% of the *Serratia* population in fish filets during storage at 6°C, linked with a bacterial count reduction in TSA and Long & Hammer agar. No Ca^2+^ additions were necessary for maximum activity, which would simplify their application in the large-scale fish-processing systems.

## Author Contributions

IH was responsible of the conception and design of the work, the acquisition, analysis and interpretation of the data presented in this work.

## Conflict of Interest Statement

The author declares that the research was conducted in the absence of any commercial or financial relationships that could be construed as a potential conflict of interest.

## Abbreviations

MOI: multiplicity of infection
Pfu/ml: plaque formation units per milliliter.
