# Supplementary material for: Bacteriophages against Serratia as Fish Spoilage Control Technology
Source: Front Microbiol. 2017 Apr 4;8:449. doi: 10.3389/fmicb.2017.00449 (PMC5378772; doi:10.3389/fmicb.2017.00449)
Supplement: Supplementary file 1 [file Data_Sheet_1.pdf]

Supplementary Material

Bacteriophages against *Serratia* as Fish Spoilage Control Technology

Igor Hernández*

E- mail address: ihernandez@azti.es

Supplementary Table 1. Maximal bacteriophage titration (Log pfu/ml) after a single cultivation round (SFO009 as target strain)

Phage	Max titration
AZT1	9.3
AZT2	8.7
AZT3	8.8
AZT4	7.3
AZT5	7.8
AZT6	8.2
AZT7	7.0
AZT8	7.5
AZT9	6.9
AZT10	7.1

Supplementary Table 2. Bacteriophage titration reduction (log cfu/ml) after storage at -80 ºC (7 days) or 4 ºC (30 days).

Phage	Reduction	
	-80 ºC	4ºC
AZT1	-1.8	-1.7
AZT2	-5.2	-4.8
AZT3	-1.3	-1.3
AZT4	-1.4	-1
AZT5	-0.9	-1.3
AZT6	-0.6	-0.9
AZT7	-1.4	-1.2
AZT8	-5.3	-4.7
AZT9	-1.8	-2.0
AZT10	-1.9	-1.7

Supplementary Figure 1. Bacteria growth (SFO009) in presence of bacteriophage (blue line, in all cases MOI was 1:1) or bacteriophage + 10mM Ca^{+2} (red line). AZT1 (A), AZT2 (B), AZT3 (C), AZT4 (D), AZT5 (E), AZT6 (F), AZT7 (G), AZT8 (H), AZT9 (I) AZT10 (J) and control without bacteriophage (K).

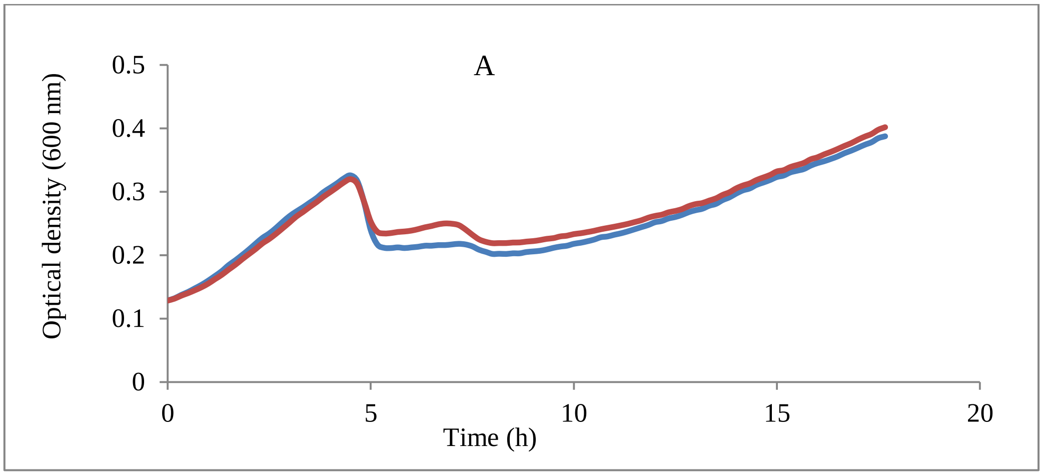

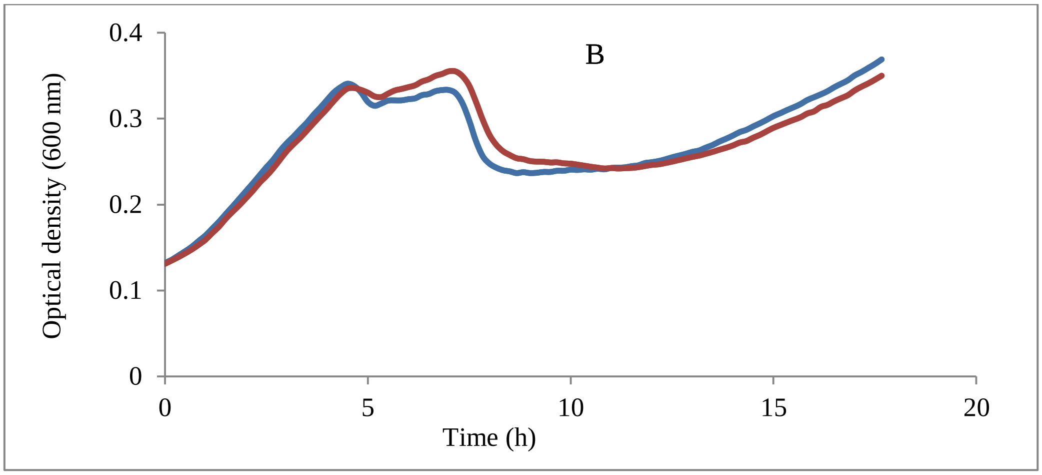
B
Optical density (600 nm)
Time (h)
0.4
0.3
0.2
0.1
0
0
5
10
15
20

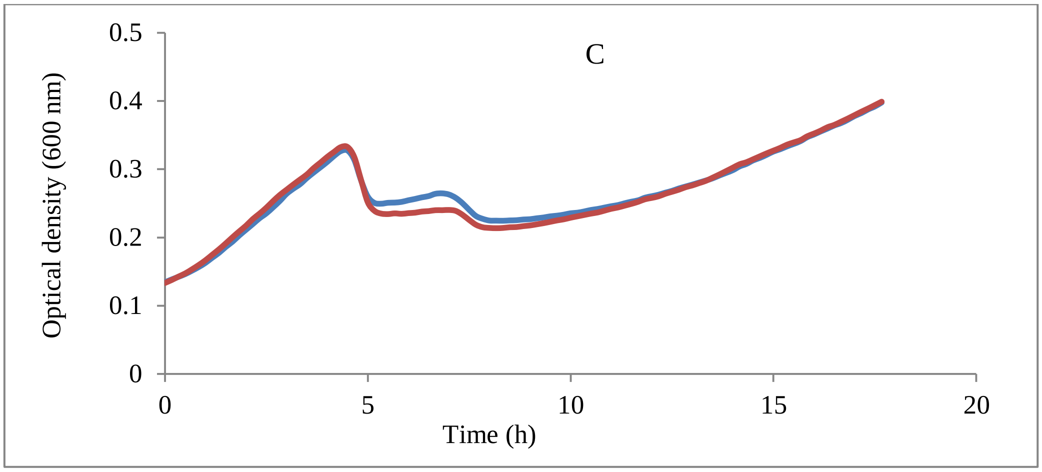
C
Optical density (600 nm)
Time (h)
0.5
0.4
0.3
0.2
0.1
0
0
5
10
15
20

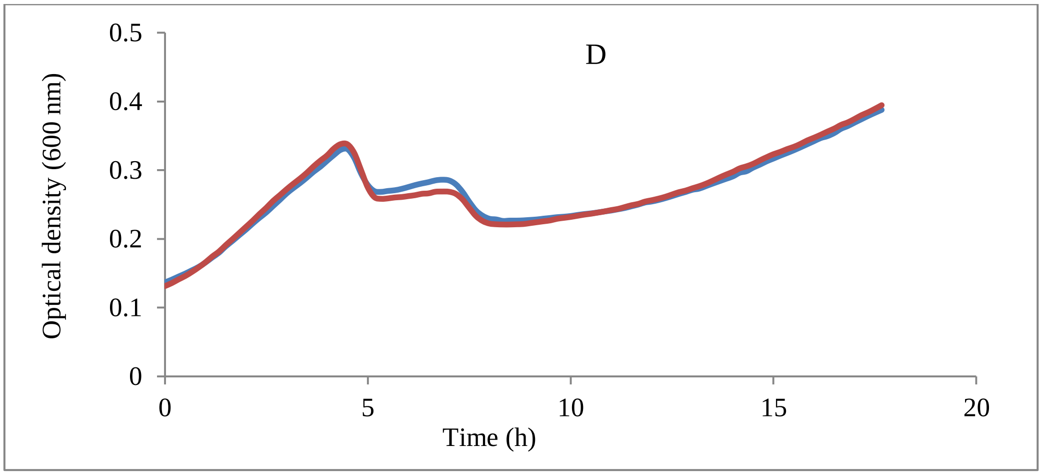
D
Optical density (600 nm)
Time (h)
0.5
0.4
0.3
0.2
0.1
0
0
5
10
15
20

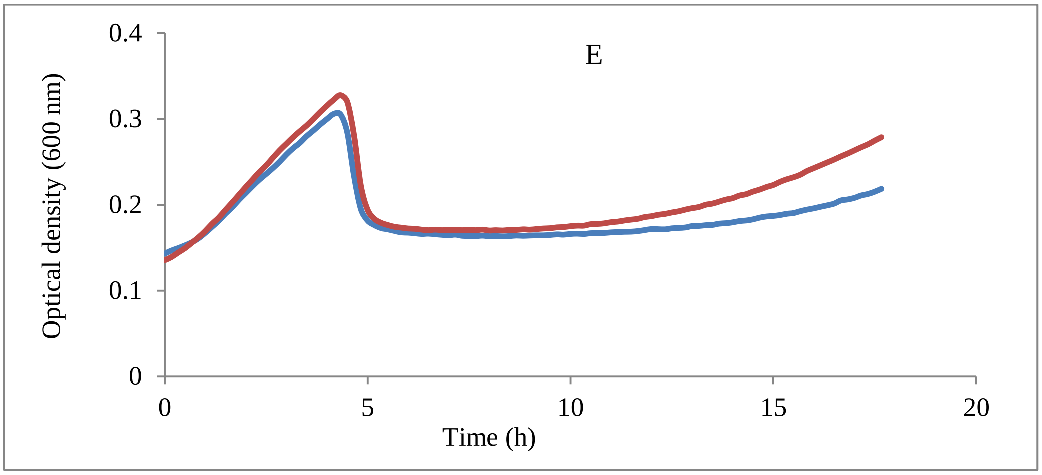
E
Optical density (600 nm)
0.4
0.3
0.2
0.1
0
0
5
10
15
20
Time (h)

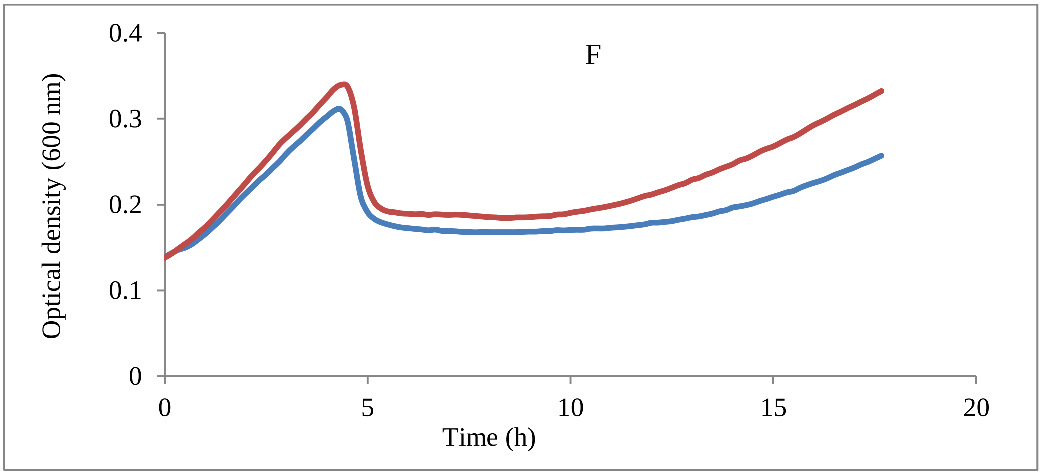
F
Optical density (600 nm)
0.4
0.3
0.2
0.1
0
0
5
10
15
20
Time (h)

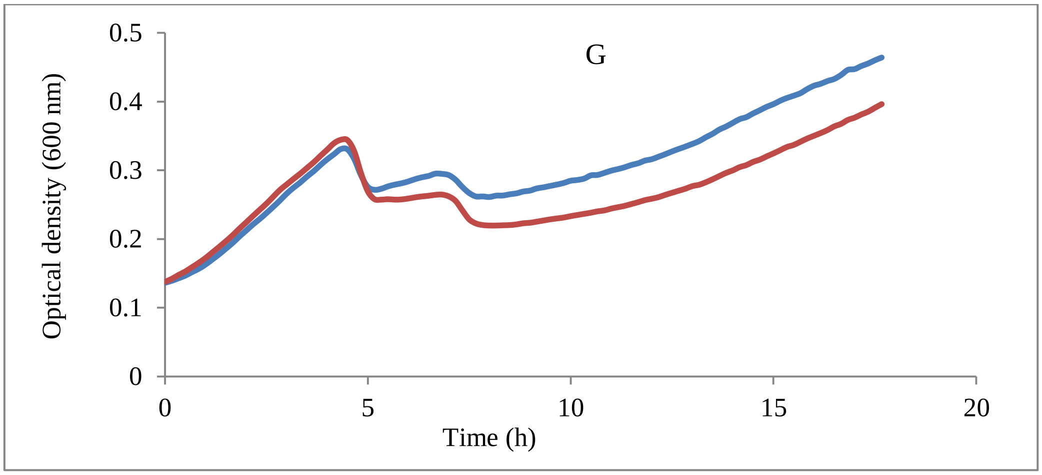
G
Optical density (600 nm)
0.5
0.4
0.3
0.2
0.1
0
0
5
10
15
20
Time (h)

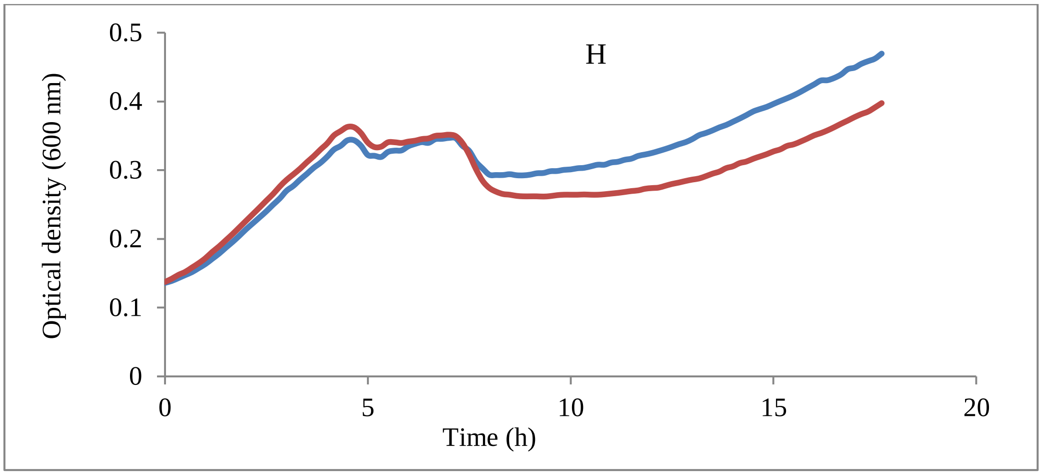
H
Optical density (600 nm)
0.5
0.4
0.3
0.2
0.1
0
0
5
10
15
20
Time (h)

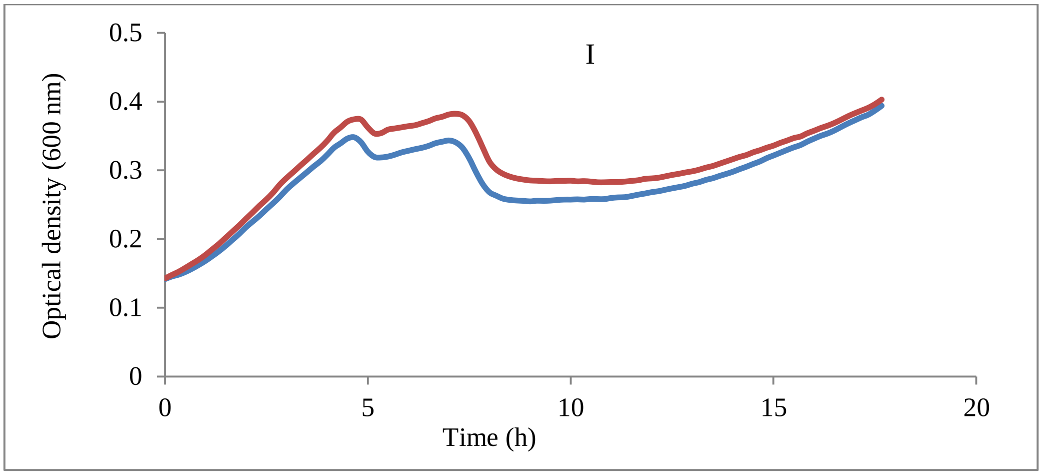
I
Optical density (600 nm)
0.5
0.4
0.3
0.2
0.1
0
0
5
10
15
20
Time (h)

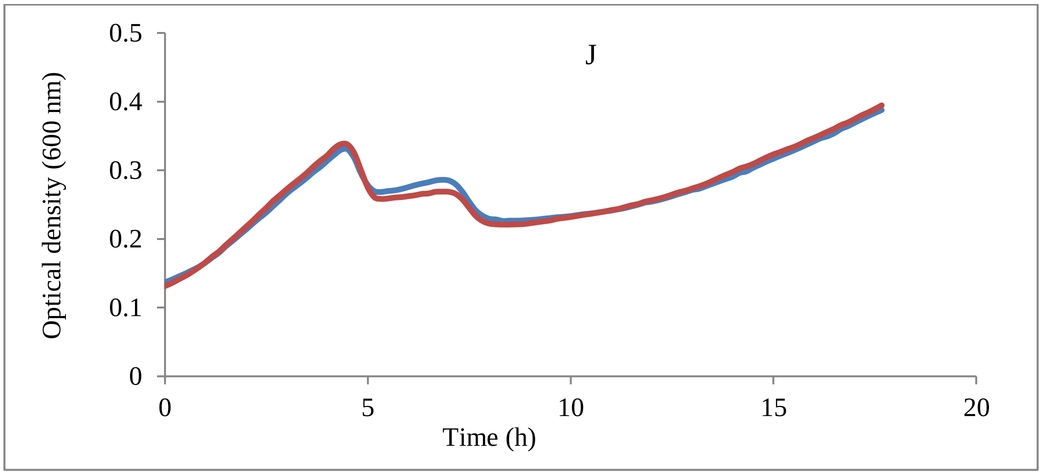
J
Optical density (600 nm)
0.5
0.4
0.3
0.2
0.1
0
0
5
10
15
20
Time (h)

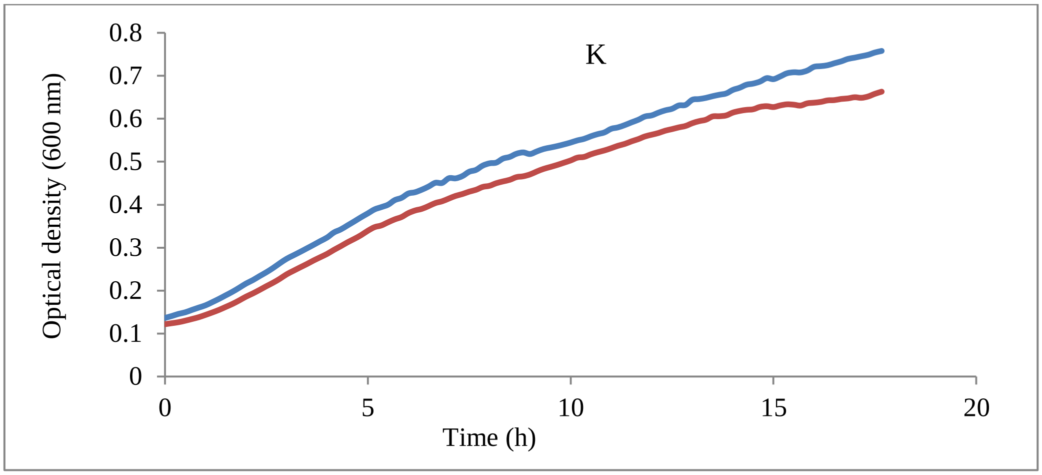
K
Optical density (600 nm)
0.8
0.7
0.6
0.5
0.4
0.3
0.2
0.1
0
0
5
10
15
20
Time (h)
